# Accuracy of ultrasonography in the diagnosis of acute appendicitis in adult patients: review of the literature

**DOI:** 10.1186/2036-7902-5-S1-S2

**Published:** 2013-07-15

**Authors:** Fabio Pinto, Antonio Pinto, Anna Russo, Francesco Coppolino, Renata Bracale, Paolo Fonio, Luca Macarini, Melchiorre Giganti

**Affiliations:** 1 Department of Diagnostic Imaging, Marcianise Hospital, ASL Caserta (CE), Caserta, Italy; 2 Department of Diagnostic Imaging, A. Cardarelli Hospital, Naples, Italy; 3 Department of Radiology, S G. Moscati Hospital, Aversa, Italy; 4 Department of Radiology, University of Palermo, Palermo, Italy; 5 Department of Health Science, University of Molise, Campobasso, Italy; 6 University of Turin, Institute of Diagnostic and Interventional Radiology, Turin Italy; 7 Department of Radiology, University of Foggia, Foggia, Italy; 8 University of Ferrara, Dipartimento di Scienze Chirurgiche, Ferrara, Italy

**Keywords:** Acute appendicitis, Ultrasound, Abdomen (US), Abdominal Pain

## Abstract

**Background:**

Ultrasound is a widely used technique in the diagnosis of acute appendicitis; nevertheless, its utilization still remains controversial.

**Methods:**

The accuracy of the Ultrasound technique in the diagnosis of acute appendicitis in the adult patient, as shown in the literature, was searched for.

**Results:**

The gold standard for the diagnosis of appendicitis still remains pathologic confirmation after appendectomy. In the published literature, graded-compression Ultrasound has shown an extremely variable diagnostic accuracy in the diagnosis of acute appendicitis (sensitivity range from 44% to 100%; specificity range from 47% to 99% ). This is due to many reasons, including lack of operator skill, increased bowel gas content, obesity, anatomic variants, and limitations to explore patients with previuos laparotomies.

**Conclusions:**

Graded-compression Ultrasound still remains our first-line method in patients referred with clinically suspected acute appendicitis: nevertheless, due to variable diagnostic accuracy, individual skill is requested not only to perform a successful exam, but also in order to triage those equivocal cases that, subsequently, will have to undergo assessment by means of Computed Tomography.

## Background

Appendicitis represents one of the most common causes of abdominal pain of adult patients referred to the emergency department. More than 250,000 cases of appendicitis are diagnosed in the United States each year, and appendectomy is the most frequent emergent surgery performed worldwide [1,2]. Despite its prevalence, the diagnosis of appendicitis can be elusive and fraught with pitfalls because of the absence of a pathognomonic sign or symptom, the poor predictive value of associated laboratory testing, and its varied presentation diagnosis [3-5]. The rate of unnecessary laparotomies is still high: to balance an acceptable positive laparotomy rate with minimal delayed or missed diagnoses, the clinician must take into account all the available historical and physical findings, laboratory data, and appropriate imaging method. In fact, following significant advances in accuracy, imaging is an important part of the modern work-up of appendicitis, that remains a high-risk disease for delayed or missed diagnosis in the emergency department [6,7].

Among imaging methods currently used in the clinical practice, Ultrasound (US) is a valuable tool. It was first introduced by Puylaert in 1986, who described the "graded compression" technique apt to better visualize the inflamed appendix [8]; by using the graded compression technique, a linear high-frequency transducer is placed on the right lower quadrant and pressure is applied gradually while imaging, displacing overlying gas-filled loops of bowel. Moreover, this noninvasive option is repeatable, avoids the exposure to nonionizing radiation and can be less expensive as compared to Computed Tomography (CT) costs. At US, findings suggestive of appendicitis include, a thickened wall, a noncompressible lumen, outer appendiceal diameter greater than 6 mm, absence of gas in the lumen, appendicoliths, echogenic inflammatory periappendiceal fat change, and increased blood flow in the appendiceal wall . If compared to other diagnostic tests, US is inferior to CT as to sensitivity; due to its low negative predictive value for appendicitis, it may not be as useful for excluding appendicitis. More recently, color and power Doppler examination of the appendix have proven to be a useful adjunct to improve the sensitivity by demonstrating increased flow in an inflamed appendix [9,10].

Indeed, US is not accepted worldwide to rule out an acutely inflamed appendix: the quality of the ultrasound examination improves with operator experience and skill. Accordingly, the purpose of this study was to investigate the diagnostic accuracy of the US method in the diagnosis of acute appendicitis of the adult patient as in the literature reported [11,12].

## Results and discussion

Although US is frequently used to diagnose acute appendicitis, the accuracy of this imaging test remains unclear because of a great variability in the reported performance. An evidence-based review of the role of graded compression US for the diagnosis of appendicitis was performed by Terasawa and coworkers [13]: they found that 14 studies of graded compression US could meet their inclusion criteria: Ultrasonography showed an overall sensitivity of 0.86 and a specificity of 0.81, a positive predictive value of 84%, and a negative predictive value of 85%.

In Korea, a large meta-analysis on the role of graded compression US in the diagnosis of acute appendicitis was carried out a few years ago, including 22 articles [14]. The overall sensitivity and specificity were 86.7% and 90.0%, respectively. In particular, their study suggested that US could be useful for the diagnosis of acute appendicitis, especially when patients were younger age, male, and highly clinical suggestive.

In other published series, overall sensitivity of US in adult and adolescent patients was 86%, specificity 81% , the positive predictive value of graded compression US was 84% (range from 46% to 95%), and the negative predictive value of graded compression US was 85% (range from 60% to 97). While the range of reported accuracy (82% to 96%) for US in children has been acceptable, the sensitivity (44% to 100%) and the specificity (47% to 99%) have varied considerably; also, the visualization rates vary widely in the published literature, from a low of 22% to a high of 98% [14]. Several factors might be taken into account as the causes of these variations. First, because US is an operator-dependent technique, with a steep learning curve, individual skill may be an important factor to determine an extremely variable diagnostic accuracy of appendicitis [15]. Moreover, difficulties to scan populations of fertile age females may be related to the broad and frequent overlap of the symptoms for acute abdominal conditions [16-20]. In obese patients, as well in individuals who underwent previous laparotomy, adequate compression of the right lower quadrant, according to the graded compression technique, cannot be always obtained. Variability in the appendiceal location is a well known cause for clinical misdiagnosis, and a false negative US diagnosis may occur, for example, in case of a retrocecal location of the appendix, not appropriately visualized. Indeed, most of the false-negative diagnoses at US result from non-visualization of the appendix or from inflammation limited to the appendiceal tip [15-17]. While positive ultrasound findings have a relatively high positive-predictive value, identification of a normal appendix is sometimes difficult. Excellent results have been achieved at select centers, with nonvisualization of the appendix being reported to have a negative-predictive value of 90% [21]. Such results require a great deal of skill and experience; in fact, in many centers nonvisualization of the appendix is considered equivocal.

## Conclusions

Imaging is necessary in adult patients referred with clinically suspected acute appendicitis: in fact, there is wide agreement that the outcome of acute appendicitis is best with early diagnosis. Graded-compression US remains our first-line method in the evaluation of patients referred with clinically suspected acute appendicitis. It can be performed at any time, regardless of specific patient's preparation. Nevertheless, due to variable diagnostic accuracy, individual skill is requested not only to perform a successful exam, but also to triage those equivocal cases that, subsequently, will have to undergo Computed Tomography assessment [22,23].

## Competing interests

All the authors declare that they have no competing interests.

## Declarations

This article has been published as part of *Critical Ultrasound Journal* Volume 5 Supplement 1, 2013: Topics in emergency abdominal ultrasonography. The full contents of the supplement are available online at http://www.criticalultrasoundjournal.com/supplements/5/S1. Publication of this supplement has been funded by the University of Molise, University of Siena, University of Cagliari, University of Ferrara and University of Turin.
